# Dental Furnaces

**Published:** 1895-05

**Authors:** W. M. Sharp

**Affiliations:** Binghampton, N. Y.


### DENTAL FURNACES.[1]

BY DR. W. M. SHARP, BINGHAMPTON, N. Y.

Gentlemen,—The subject of my paper this evening is a matter which ought to concern every practitioner of dentistry. It is safe, however, to state that a very small percentage are acquainted either in the construction of a furnace or its use.

If one should go on a tour of inspection through the dental offices of to-day, he would find a large number equipped in the manner I will attempt to describe.

In our profession we have classified our operations, and have named the one prosthetic, the other operative, the operations being equally important; and in our offices we have prepared rooms for each of these.

In the operating-room will be found either an oiled floor, or a beautifully carpeted room, pretty curtains, rugs, handsome furniture, the most modern operating-chair, all the electrical appliances, a dental cabinet that has within it an assortment almost equal to a dental depot,—innumerable instruments, excavators, elevators,

---

[1] Read before the Odontological Society of Pennsylvania, December 8, 1894.

illuminators, generators, and regulators; rubber-dam clamps for the proximal, distal, labial, lingual, and cervical portion of every tooth. Theoretically these are all perfect and necessary; practically a much smaller and well-selected assortment of instruments would meet all requirements. In such an operating-room there is no indication of superior mechanical ability; there is evidence that some one else has done the originating, and it only remains for the operator to study their application. In fact, everything of comfort and convenience may be found in this room to facilitate the success of the operation.

The mechanical room presents an entirely different aspect. Any room in the building is considered good enough for this purpose. A back room where there is but little light will answer. White walls, bare windows, cold, no carpet. In one part of the room there is a lathe, in another a plaster-can, some plaster in it and a good deal on the floor, a bench or table covered with wax, impression-trays, scrapers, and old broken plaster models. On a table or in one corner there is standing on guard a vulcanizer. It looks well in this room, is strong, and would be stronger if it did not leak steam. With the exception of a few dishes and bottles, this is pretty nearly all that will be found. There is nothing inviting in the room; there is no interest; how could there be under such circumstances? In the office the very instruments of the operating-room have the look of superiority over those of the mechanical, while the mechanical instruments have the look of insignificance and have not the power to assert their importance; they are deficient in numbers and inferior in quality. Certainly there is a marked contrast of the one over that of the other. What has occasioned this lack of interest in this branch? for it was never intended that any part of our profession should be neglected, but there should be perfect harmony and an increased interest in all operations.

A little investigation and the cause is easily traced. Unfortunately some people have a most contemptible element in their nature. It is this feeling of superiority over his fellow, and a belittling of others and their vocations, where self-esteem knows no bound. In our profession we have a few of these people. They would have their patients understand that there is a vast difference in the operations of dentistry; that the care of natural teeth requires greater skill than does the mechanical operations. There are so many objectionable duties connected with that branch, and he prefers to let some one else do it, while he is entirely an operative dentist; he is working to give his patient the impression that he is in advance of the ordinary dentist. There are a good many operators of this class who have this feeling towards mechanical dentistry.

Certainly there are exceptions. Some have taken it up as a specialty; there can be no point raised against any man for making a specialty of any part of the profession; but when this is done, it never occurs to him to feel himself above other members no matter what success he may have in it.

There can be no doubt but this existing feeling has to a considerable extent interfered with the interests that would otherwise have been in this branch.

The principle reason, however, I think is due to thoughtlessness on the part of the dentist in not having more pleasant surroundings, more of the comforts and conveniences in this room. If one is a mechanic, which he must be to successfully practise this profession, he will feel more at home in this work than at operating. If any man should feel himself superior to another, the mechanical man has far more reasons for doing so. One single successful operation of an artificial palate gives a dentist a standing that could not be reached in operative dentistry. The same may be said of an artificial nose, artificial ears, artificial teeth. The smallest fee charged for a mechanical operation is far in advance of the smallest in operative dentistry.

As regards the unpleasant duties in mechanical dentistry, there are none. If they refer to a little plaster of Paris on one's fingers occasionally, I can only reply, I would rather have my hands covered with plaster in connection with a case for which I was to receive one hundred dollars than I would to work over a nervous patient at the chair, the equivalent of time, on the treatment of putrescent pulps, and for which I would not receive more than one-fifth this amount.

Now, let us all elevate mechanical dentistry and attempt more difficult operations. There is a wide field for investigation and improvement in methods and appliances and possibilities for greater attainments. Once interested there is no greater fascination.

My idea of a laboratory is a room which is conveniently located, pleasant, properly lighted and ventilated, and windows trimmed, not necessarily with expensive material, comfortable chairs, carpet on the floor. I use Scotch cork carpet: this is noiseless and can be mopped, and adds much to the room. For furniture, this may be largely in the shape of cabinets for instruments and tables of operations. Don't buy cheap things; get something you like and you will always have an interest in it.

Be careful in your selection of instruments and appliances when you get them. No man can make a proper selection of instruments if he is not first familiar with the operation he is to perform; if he is, it takes but little explanation of the working of any apparatus for him to decide promptly whether or not it will be valuable in assisting him in his operations. If you think an instrument will be valuable, procure it; if you think it will not be valuable, do not get it because the dealer or agent says some popular dentist has one. It may be that his idea of instruments and yours differ. What would be successful in his hands might prove detrimental in yours; and because your results were not equal to his you would in all probability charge your failures to the apparatus, never stopping to consider the fault is with yourself. In the first place, perhaps your knowledge on this subject is not quite as good, and that in trying to manage something that you are not satisfied with you lose interest. Fortunately we have a variety of appliances for making our artificial substitutes differently constructed but reaching the same end, which gives us a chance to choose that which is best adapted to meet the requirements.

Now, with care one may have his laboratory fitted out with appliances that are constantly in use. We will assume he has made a good selection of everything except a furnace. He will need something by which he can quickly and conveniently melt his zinc, lead, or to refine metals, to prepare his cases for soldering, to make a porcelain crown which he has carved for some special case, to make an aluminum plate, to make a porcelain bridge, or for the repair of porcelain, or to bake the teeth which he has carved for special cases, and to make continuous-gum teeth. For each of these operations he will require heat. Since in our variety of dental furnaces there is made a separate one for each of the above cases, it might be thought necessary by the inexperienced to purchase one of each kind so that he would be fully equipped. In my judgment I do not think this necessary.

The most desirable is that which is so constructed that it will make laboratory work light and convenient, and will give opportunities for the greatest range of uses in the shortest possible time.

The most desirable fuel will be that which is most prompt in action, the least objectionable to work with, and which is controlled without fear of endangering one's self in its use, since the results will be the same when the desired temperature is reached.

The success of the operation will therefore depend upon the construction of the furnace, together with the manner in which it is operated, with little reference to the combustible used. In this direction I recommend your investigation in selecting your furnace.

The study of combustion will settle this question for you and fit you to scrutinize to your entire satisfaction the most complicated furnace.

Except for use in connection with porcelain work, there is practically no need of inquiring into detailed construction, for it matters not whether they have been built with a view to having perfect combustion or not, if only the temperature can be reached and they are otherwise satisfactory.

Porcelain is highly sensitive to certain gases and to foreign matter, so that there is provided a chamber or muffle which protects it from the direct influences of the fuel used. Cases injured from this cause are termed gased.

Let us consider the furnace which has been in use the longest time and is in some respects the best. It is the large coal furnace which is in every college building and with which you are familiar. In the centre of this is a large space for coal, with a clay muffle stretching across from one side to the other. The great amount of coal or coke surrounding the muffle furnishes a steady volume of heat which is necessary to get the best results in porcelain work. No better work has ever been done than that made in this style of furnace when the management has been good. But the inexperienced will gas his work every time, until he learns that he must attend to the draughts and get his fire in proper shape before he commences his baking. If one is doing a great amount of this work, sufficient to keep it running a full day at a time, and has plenty of room to keep it in and is indifferent to the temperature of his room while working, this will do the work perfectly.

For me to go into detail on the construction of the different furnaces now in use for this purpose, it would seem unnecessary, since I have made a careful study of the underlying principles and have endeavored to present them as they appear to me, and since I have brought with me a furnace of my own construction which will convey to you my ideas on this subject better than I could otherwise present them.

